# Supplementary material for: MetaRibo-Seq measures translation in microbiomes
Source: Nat Commun. 2020 Jun 29;11:3268. doi: 10.1038/s41467-020-17081-z (PMC7324362; doi:10.1038/s41467-020-17081-z)
Supplement: Supplementary file 10 — Supplementary Data 7 [file 41467_2020_17081_MOESM10_ESM.zip › File2/Confidence_VeryHigh_Taxonomy/117104_out.krona.html]

Javascript must be enabled to view this page.

members
magnitude
magnitudeUnassigned
count
unassigned
taxon
rank

117104\_out

4

superkingdom
2
4

4
1239
phylum

4
class
186801

4
order
186802

family
186803
1

1
genus
189330

species
88431

SRS893295\_contig\_number\_contig-100\_37621.37622
1

1
family
186807

1562
genus
1

species
2305243

SRS1041133\_contig\_number\_contig-100\_2755.133907
1

family
186806
2

genus
1730
2

1262892

SRS024075\_contig\_number\_contig-100\_1526.110412
species
1


SRS077454\_contig\_number\_contig-100\_3922.123695
39485
species
1
